# Natural and Synthetic Halogenated Amino Acids—Structural and Bioactive Features in Antimicrobial Peptides and Peptidomimetics

**DOI:** 10.3390/molecules26237401

**Published:** 2021-12-06

**Authors:** Mario Mardirossian, Marina Rubini, Mauro F. A. Adamo, Marco Scocchi, Michele Saviano, Alessandro Tossi, Renato Gennaro, Andrea Caporale

**Affiliations:** 1 Department of Medicine, Surgery and Health Sciences, University of Trieste, Piazza dell’Ospitale, 1, 34125 Trieste, Italy; 2 School of Chemistry, University College Dublin, Belfield, Dublin 4, Ireland; marina.rubini@ucd.ie; 3 Department of Chemistry, Centre for Synthesis and Chemical Biology (CSCB), RCSI, 123 St. Stephens Green, Dublin 2, Ireland; madamo@rcsi.ie; 4 Department of Life Sciences, University of Trieste, Via L. Giorgieri, 5, Q Building, 34127 Trieste, Italy; mscocchi@units.it (M.S.); atossi@units.it (A.T.); rgennaro@units.it (R.G.); 5 Institute of Crystallography (IC), National Research Council (CNR), Via Amendola, 122, 70126 Bari, Italy; michele.saviano@ic.cnr.it; 6 Institute of Crystallography (IC), National Research Council (CNR), c/o Area Science Park, S.S. 14 Km 163.5, Basovizza, 34149 Trieste, Italy

**Keywords:** antimicrobial peptides (AMPs), structure-activity relationship, fluoro amino acids, fluoro-proline, halogenation, bromo-tryptophan, peptides, α- and β-peptoids

## Abstract

The 3D structure and surface characteristics of proteins and peptides are crucial for interactions with receptors or ligands and can be modified to some extent to modulate their biological roles and pharmacological activities. The introduction of halogen atoms on the side-chains of amino acids is a powerful tool for effecting this type of tuning, influencing both the physico-chemical and structural properties of the modified polypeptides, helping to first dissect and then rationally modify features that affect their mode of action. This review provides examples of the influence of different types of halogenation in amino acids that replace native residues in proteins and peptides. Examples of synthetic strategies for obtaining halogenated amino acids are also provided, focusing on some representative compounds and their biological effects. The role of halogenation in native and designed antimicrobial peptides (AMPs) and their mimetics is then discussed. These are in the spotlight for the development of new antimicrobial drugs to counter the rise of antibiotic-resistant pathogens. AMPs represent an interesting model to study the role that natural halogenation has on their mode of action and also to understand how artificially halogenated residues can be used to rationally modify and optimize AMPs for pharmaceutical purposes.

## 1. Introduction

**Figure 1:**
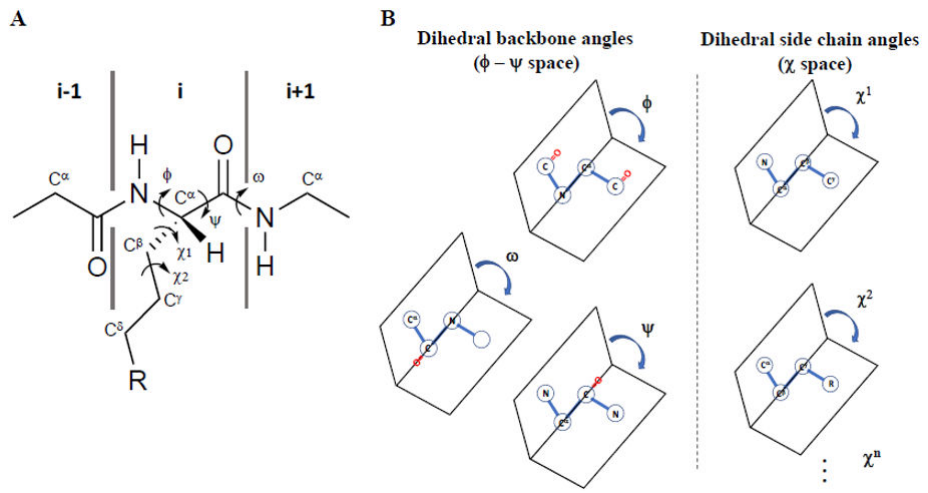
(**A**) Representation of the φ, ψ, ω, χ^1^, and χ^2^ torsional angles in a peptide, and (**B**) their definition as dihedral angles in 3D space, adapted from Ref. [4].

Since 1997, Prof. Victor Hruby has stressed how three-dimensional structure can influence the biological activities of peptides and proteins both in the free state and when engaged in interaction with their receptors/acceptors. If, on one hand, the conformations of the backbone (α-helix, β-sheet, β-turn etc.) are the main determinants for three-dimensional structures, on the other, the arrangement of side chains in three-dimensional χ space (where χ^1^, χ^2^ etc. are the torsional angles of the side chain bonds) are determinant for molecular recognition, signal transduction, enzymatic specificity, immunomodulation, and other biological effects (Figure 1A). Using computational and experimental methods, it is possible to examine the effects of specific structural modifications in constraining the side chain groups of amino acid residues, or of their mimetics, in χ space [1]. Moreover, to study the physico-chemical features required for the design of novel peptide and peptidomimetic agonists, antagonists, inverse agonists, a special emphasis must be given to the use of conformational (ϕ-ψ space) and topographical (χ space) considerations [2,3] (Figure 1B). 

The present work reviews the structural features induced by the presence of halogens on residue side-chains and the role of the halogen itself as well as of the synthesis and application of halogenated amino acids. In nature, halogenated amino acids are quite rare and require enzymes to introduce the halogen as a post-translational modification. Non-proteinogenic amino acids may have diverse physiological functions, for example, regulatory functions in metabolic cycles [5], or anti-bacterial, anti-inflammatory, anti-tumor and anti-hypertensive effects. Extensive reviews have already been published on these aspects [6,7,8]. The properties of halogenated antimicrobial peptides (AMPs), both native and synthetic, and their possible contribution to the growing problem of antibiotic resistance are also considered. 

## 2. The Structural and Physico-Chemical Effects of Halogen Atoms in Polypeptides

The introduction of halogen atoms provides many more benefits than other types of substitutions in drugs [9]. In fact, it is estimated that up to one third of drugs undergoing clinical investigation is halogenated [10]. This type of substitution can be used to control the degradability of pharmaceuticals as well as their lipophilicity, membrane permeabilization activity and catabolic stability [11,12]. The ligand-receptor interaction can be modulated by the presence of halogen atoms because of their electron-withdrawing features and their steric-effects [10,13]. This can interestingly find applications from very small molecules to large complexes such as antibodies. For example, halogenated Tyr improved 10 fold the binding affinity of the Fab fragment of an anti-EGF-receptor (059-152) for its antigen [14].

In this role, the halogen atom can also act as an electron donor by allowing the formation of stabilizing interactions such as hydrogen bonds [15,16]. Although it is hydrophobic in nature, the halogen introduces some interesting physico-chemical features not normally associated with this property, such as allowing for the possibility of F···H–N hydrogen bond formation. This is confirmed by through-space coupling in NMR studies [17,18].

The introduction of fluorine into a peptide or a protein offers rich opportunities both when the fluorination site is on an amino acid side chain or on the backbone itself, because of its capacity to locally alter the electronic character of the peptide/protein [19]. The fluorination also has an effect on the folding properties, changing the secondary structure propensity of peptides and protein segments containing aliphatic halogenated amino acids. In addition, fluorine has an impact on the proteolytic stability of peptides, in a manner that depends both on the nature of the fluorinated side chain but also on its effect on the immediate environment [20]. Resistance towards proteolytic digestion due to the introduction of halogenated amino acids cannot however be generalized. It depends on the substitution position and number of introduced halogen atoms on the amino acid side chain. For example, a minimal presence of fluorinated, small side chain amino acids can improve remarkably proteolytic stability [12]. Futhermore, bulky fluorinated residues, such as hexafluoroleucine or trifluoroisoleucine protect polypeptides from proteolytic enzymes. On the other side, fluorinated aromatic amino acids are comparable to their hydrocarbon counterparts in maintaining the proteolytic stability [12]. These observations are rendered even more interesting by studies showing that the introduction of fluorine is relatively non-invasive with respect to structural stability. For example, Kovermann and co-workers investigated the thermodynamic stability and structural integrity of Cold shock protein B from Bacillus subtilis (BsCspB), containing fluorine-labelled phenylalanine or tryptophan residues. Time-resolved fluorescence kinetics was used to monitor chemical denaturation of fluorine-labelled BsCspB, while thermal denaturation was followed by high-resolution ^1^H and ^19^F NMR, X-ray [21]. These experiments showed that the presence of F-Phe or F-Trp residue caused only a barely detectable change in thermodynamic stability in comparison to the wild type protein. Moreover, the X-ray structures of fluorinated CBsCspB variants were almost completely superposable with the structure of the wild type protein.

However, since the alignment of C-F bonds with adjacent C=O or C-N bonds is predictable, fluorinated amino acids might be used as a tool to study and control the secondary structure of polypeptides [22]. In this context, 4-fluoroprolines are excellent building blocks to study the pharmacological and structural impact caused by their substitution, especially for the stability and kinetics of protein folding. In this context, the rational substitution of Pro or Hpy with a 4-fluorinated Pro analogue in peptides and proteins can enhance both the thermodynamic and hydrolytic stability, as well as the resistance to unfolding in organic solvents of the engineered protein [23,24]. 

**Figure 2:**
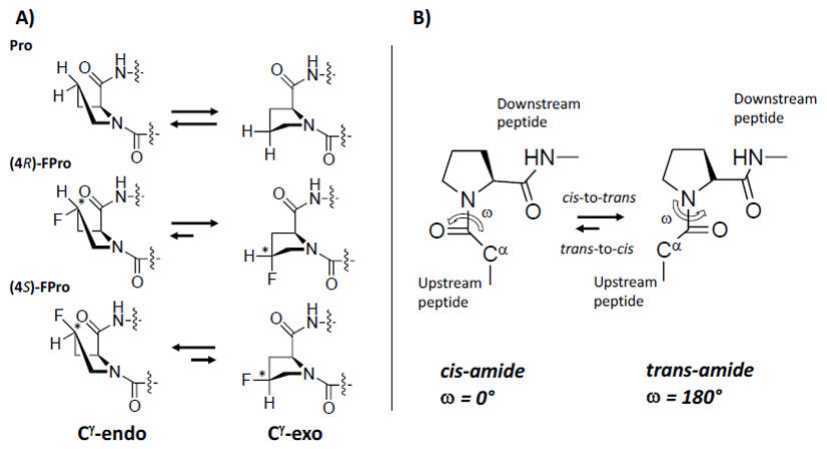
Fluorine effect on proline conformation. (**A**) pyrrolidine ring puckers in proline, (4R)-FPro, and (4S)-FPro. Fluorine on the C^γ^ atom introduces a chiral center (indicated by *) with effect on the preference of endo/exo-pucker. (**B**) cis/trans isomerization by Pro. Figure adapted from Ref. [32].

The pyrrolidine ring of proline introduces a conformational rigidity and constrains into polypeptide backbone, as two of the main chain atoms are constricted on the ring [25,26,27,28]. The pyrrolidine ring adopts predominantly two different conformations. In the endo conformation (C^γ^-endo), carbon 4 and α-carbonyl carbon are on the same side of the plane, while in the exo conformation (C^γ^-exo), carbon 4 on the pyrrolidine ring points towards the opposite side of the carbonyl group of Pro (Figure 2). The presence of fluorine on the C^γ^ atom, with its electron-withdrawing properties, can bias the proline ring pucker preferences and “pre-organize” the protein main chain, thus conferring additional stabilization to the whole protein [29,30]. Thus, (2S, 4S)-4-fluoroproline ((4S)-FPro), prefers the C^γ^-endo ring pucker and stabilizes the cis peptide-bond conformation, while (2S, 4R)-4-fluoroproline, ((4R)-FPro) stabilizes C^γ^-exo ring pucker favoring the trans conformation [28,31,32] (Figure 2). 

In folded polypeptides, Xaa-Pro (Xaa = any amino acid) peptide bonds still display preferentially the trans conformation, although in this case both conformations are almost isoenergetic [33]. The cis/trans isomerization of Xaa-Pro bonds is critical during the folding process in polypeptides. The refolding of globular proteins presents an intrinsically slow isomerization step when non-native trans prolyl-peptide bonds in the unfolded state undergo isomerization to reach their native cis state [25]. Therefore, the cis/trans isomerization reaction is typically the rate-limiting step in the native protein folding or refolding processes. The substitution of 10 prolines (9 trans and 1 cis) in the Enhanced Green Fluorescent Protein (EGFP) with (4S)-FPro promoted a faster refolding [25]. However, these results were attributed to several synergistic effects, arising mostly from the adoption of the endo pucker in 9 out of 10 Pro residues, rather than from cis/trans isomerization of prolines. 

**Figure 3:**
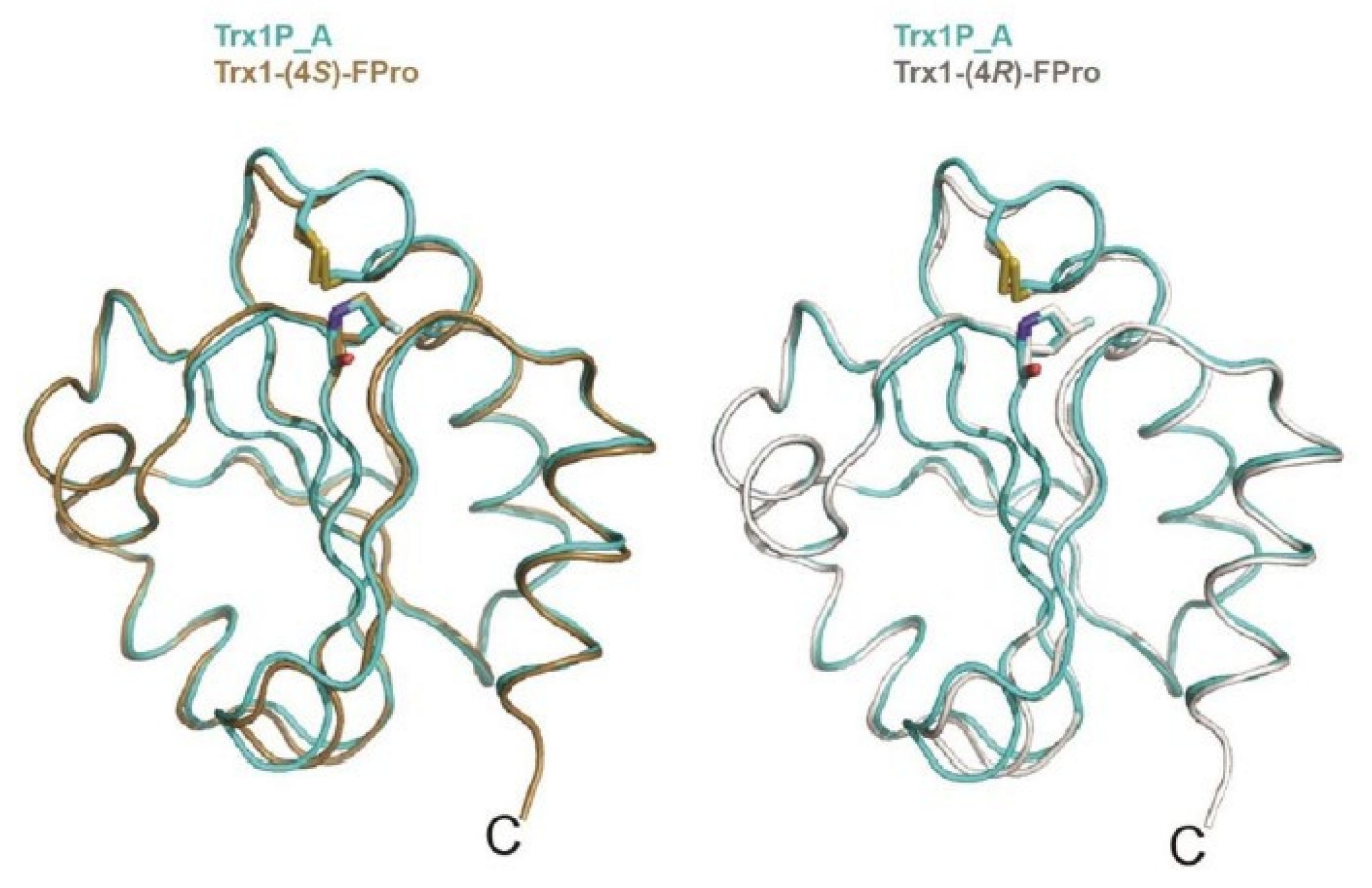
Overlay of chain A of Trx1P (cyan) on oxidized chain A of Trx1P-(4R)-FPro (light gray), on the right, and chain A of Trx1P-(4S)-FPro (brown), on the left. The cis-Pro76, cis-(4R)-FPro and cis-(4S)-FPro residues and the side chains of Cys32 and Cys35 forming the catalytic disulfide bond are shown in sticks, adapted from Ref. [36].

In 2011, Rubini and co-workers showed that the replacement of Pro 19, Pro 37, and Pro 38 (all displaying an exo pucker and all involved in trans peptide bonds) in human ubiquitin with (4R)-FPro led to the generation of a protein that was 4.71 kJ·mol^−1^ more stable than the parent protein. Moreover, the introduction of (4R)-FPro did not affect the folding mechanism or biological activity [26]. Recently, Rubini and co-workers studied the impact of 4- and 4,4-difluorinated proline analogues on thioredoxin refolding kinetics. The replacement of the conserved cis Pro76 in the E. coli thioredoxin variant Trx1P with (4S)-FPro [27] led to a 9-fold acceleration of the rate-limiting folding step, i.e. cis/trans isomerization of Xaa-Pro bond. This step occurs in the context of a long-lived folding intermediate (I_trans_) that displays an intact tertiary structure with a buried non-native trans Pro76 [34]. Interestingly, the Pro to (4R)-FPro [27] and 4,4-difluoroproline (4,4-FPro) [35] substitution at position 76 in Trx1P did not lead to an increase in the refolding rate (Table 1). The high-resolution crystal structures of Trx1P and of its monofluorinated variants (Figure 3) could not explain the difference in the refolding kinetics, as the three structures are almost indistinguishable [36].

A possible explanation for the folding behavior of these fluorinated protein variants could lie in the transition state of the refolding reaction, instead of in the 3D structure of the native state. The incorporation of (4*S*)- and (4*R*)-FPro at *cis* Pro48 led to similar results for pseudo-wild type barstar protein [37]. Similarly, to Trx1P, the *trans* to *cis* isomerization around Tyr47-Pro48 in pseudo wild type barstar during refolding, occurs in the context of a structured intermediate with Pro48 in *trans* and it is the bottleneck of the refolding reaction. Incorporation of (4*S)*-FPro at *cis* Pro48 promoted a 3.5-fold acceleration of I*_trans_* to N*_cis_* (native state), whereas (4*R*)-FPro at *cis* Pro48 did not induced changes in the refolding kinetics, in comparison to the parent protein. However, incorporation of 4,4-FPro at the same position induced a 3.6-fold increase of the I*_trans_*-to-N*_cis_* folding rate. 

Torbeev and Hilvert [38] showed that the incorporation of 4,4-FPro at cis Pro32 into human β2-microglobulin eliminated the rate limiting step for the folding of this protein. The authors suggested that the strong electron-withdrawing effect of the two fluorine atoms on the Pro ring dramatically accelerated the *trans* to *cis* prolyl bond isomerization. In fact, in comparison to the 4-monofluorinated Pro isomers, 4,4-FPro shows the lowest energy barrier for *cis/trans* isomerization; however, its application for the acceleration of folding of globular proteins seems to be context-dependent.

The introduction of (4*R*)-FPro stabilized the conformation of proline-rich peptides in aqueous solution. The proline-rich sequences in proteins that adopt a polyproline type I (PP I) helical conformation are characterized by the *cis* orientation for the Xaa-Pro bond, while PP II helical segments display prolyl amide *trans* isomers. The effect of C^γ^ *exo/endo* isomerization of the Pro ring pucker influences the *cis/trans* conformer ratio of the amide Xaa-Pro bond [28,39,40,41] as recently demonstrated by NMR, X-ray and molecular dynamics (MD) studies [42]. In collagen mimetics, the substitution of (4*S*)-Hyp or (4*R*)-FPro for the Pro residues in the sequence stabilizes the C^γ^ *exo* pucker and, thus, the PP II helix conformation. On the other hand, the substitution with (4*S*)-FPro at the Yaa-Pro position caused a relative decrease in thermostability, highlighting the impact of the 4-position stereochemistry [28]. The (4*R*)-FPro enforces the *exo* ring pucker, favoring the prolyl amide *trans* isomer [28,40,43]. With respect to the placement of F-substituted residues, Lin et al. found, using short model peptides, that C-terminal stereoelectronic effects might influence the stability of PP II conformation and the PP II/PP I conversion rate more than the N-terminal effects [44]. All these investigations have demonstrated that the use of FPro is a rational tool to probe polypeptide stability, dynamics, and conformational and structural effects, although some complications were observed possibly due to the chemical and structural constraints imposed by these complex molecules.

The study of structure-activity relationships is now supported also by computational approaches. The application of molecular dynamics (MD) simulations on peptides/peptidomimetics has become an important tool. In particular, the successful prediction of the folding and dynamics of several proteins and peptides, using MD simulations, has significant applications in drug design studies [45,46,47,48]. Their performance is linked to the accuracy of the empirical force fields used in the simulations [49]. Among noncovalent interactions, that are recognized to be important, the halogen bond (or X-bond) is an intriguing tool for engineering protein–ligand interactions and for controlling the structures of proteins and nucleic acids. A structure−activity relationship study between halogenated compounds and a subtype of 5-hydroxytryptamine receptor (5-HT_7_R) is a good example [50]. The development of new computational modeling tools for X-bonds in biological molecules, has been reported based on a set of potential energy functions that describe the anisotropic electrostatic and shape properties of the halogens participating in them [51,52]. Recently, the force field has been generalized by reducing the number of variables to just one for each halogen type and by estimating the electrostatic variable through a standard restricted electrostatic potential calculation of atomic charges. The simplified force field was validated against the AMBER force field, showing that Rappè et al.’s force field [53] is more adaptable for incorporation into classical molecular mechanics/dynamics algorithms, including those commonly used to design inhibitors against therapeutic targets in medicinal chemistry and materials in biomolecular engineering. Halogenation, and in particular fluorination, can improve several features of proteins, such as thermal and proteolytic stability and/or their enzymatic activity. A study of the impact of fluorination on hydrophobicity was recently carried out using dynamics simulations, together with a new fixed-charge, atomistic force field, to quantify the changes in hydration free energy, ΔGHyd, for amino acids with alkyl side chains and with 1 to 6 -CH → -CF side chain substitutions [54]. The results underline two main contributions traceable to alteration of side chain-water interactions and of the number of backbone-water hydrogen bonds. In conclusion, in recent years, many design tools have been developed to mechanically understand how fluorination plays a role in structure-activity relationships. This knowledge can be applied downstream in several ways, ranging from the effect on the hydrophobicity of (bio) polymers, to electrostatic properties. 

## 3. Halo-Amino Acids and Halogenated Non-Ribosomal Peptides 

Most of the molecular types belonging to the halo-amino acids of natural origin contain halogens bound to sp2 carbons. The C_sp2_-X bond (X = Br, Cl) is less prone to react with nucleophiles, for example water, but also amino groups, enolates or hydroxyls, which are ubiquitously present in living matter. For this reason, the most representative examples belong to derivatives of the aromatic amino acids: tyrosine, histidine and tryptophan. Aliphatic chlorinated non-proteinogenic amino acids, which are less reactive compared to their corresponding bromides, have also been identified in nature, where both C_sp2_-Cl and C_sp3_-Cl bonds were observed. Finally, it should be noted that halo-amino acids have not only been found in peptides, but also provided a key starting material for the assembly of complex natural products [55]. 

### 3.1. Halogenated Tyrosines and Histidines

**Figure 4:**
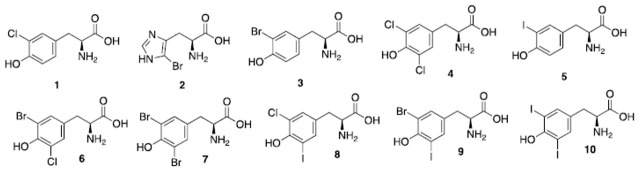
Natural halogenated tyrosines and histidine. In a recent study, tyrosines have been shown to be the preferential site of halogenation of peptides, variously leading to formation of compounds (**1**) and (**3**–**10**).

Sponges of the order *Verongida* produce bioactive halogenated amino acids. These compounds were included in the chitin-based skeletons of the demosponge *Aplysina cavernicola* (Figure 4). Specifically, 3-chlorotyrosine (**1** in the Figure 4), 5-bromohistidine (**2**), 3-bromotyrosine (**3**), 3,5-dichlorotyrosine (**4**), 3-iodotyrosine (**5*)***, 3-bromo-5-chlorotyrosine (**6**), 3,5-dibromotyrosine (**7**), 3-chloro-5-iodotyrosine (**8**), 3-bromo-5-iodotyrosine (**9**), 3,5-diiodotyrosine (**10**) were identified by electron impact (EI)-MS spectrometry in comparison with known samples [56]. The same group reported that the demosponge *Ianthella basta*, belonging to the family *Ianthellidae*, also contained a number of chlorinated, brominated and iodinated amino acids [57]. In particular, the presence of tyrosine derivatives (compounds **1**, **3**–**10** of Figure 4) was determined, while the histidine derivative (**2**) was not found in *Ianthella basta*. 3-Bromo-5-chlorotyrosine (**6**) was also previously isolated and fully characterized from the gastropod mollusk *Buccinum undatum* [58].

The compounds (**1**) and (**3**)–**)10**) were found to be variously bioactive as antibacterial, anticancer, antiparasitic, antiplasmodial, antiinflammatory, and antifouling molecules [59,60,61,62,63,64]. A recent study focused on identifying novel marine halogenated tyrosines, identified them as components of valviamides A−D (**11**–**14** in Table 2) from the cnidarians *Antipathozoanthus hickmani* and *Parazoanthus darwini* (Table 2). Antimicrobial and cytotoxicity bioassays showed that valviamide B (**12**) displayed significant cytotoxicity against the HepG2 cell line with an IC_50_ value of 7.8 μM [65]. Ascidians (sea squirts) produce an organic exoskeleton called a tunic that in *Halocynthia roretzi* has an outer thin sclerotized cuticular layer rich in proteins that include halogenated amino acids [66]. Their presence was confirmed by monoclonal antibodies specifically recognizing 3-chlorotyrosine (**1**), 3-bromotyrosine (**3**) and 3,5 dibromotyrosine (**7**) (Figure 4). 

### 3.2. Halogenated Tryptophans

**Figure 5:**
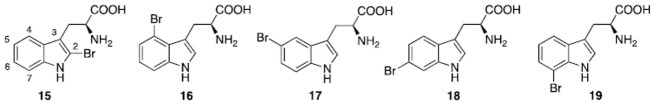
Naturally occurring halogenated tryptophans.

Five regioisomeric bromotryptophans (BrTrps) (**15**–**19** in Figure 5) have been identified in sponges and lower marine invertebrates [67]. Compounds **15**–**19**, are formed by post-translational modifications, i.e., bromination of tryphtophans after they are inserted in complex macromolecules of peptidic, macrocyclic or alkaloid nature. The relative abundance of Br in sea water (0.9 mM) and the presence of broad spectrum bromo- or lactoperoxidases in marine organisms are likely responsible of a plethora of brominated compounds in the marine flora and fauna, including peptides. All of the compounds (**15**–**19**) have been found in peptides from marine organisms. However, they have never been isolated as free amino acids, and when tested in this form for bioactivity, this was negligible.

Conversely, peptides containing the halogenated amino acids (**15**–**19**) showed significant pharmacological activities. Notably, 2-BrTrp (**15**) is present in cyclic peptides isolated from marine sponges and endowed with potent bioactivity. For example, cyclic peptides Orbiculamides [68], Keramide H [69], Kombamide [70] and Jaspamide [71] (see Table 2), are believed to have an antimicrobial role in nature and each of them demonstrated a wide range of bioactivities. 

The Styelins, broad-spectrum AMPs from the solitary tunicate *Styela clava* (see below, halogenated AMPs) are an example of peptides containing 6-BrTrp (**18**) [72]. The compound 6-BrTrp (**18**) has also been identified in conotoxins, peptides present in the venom of carnivorous marine cone snails (genus *Conus*) [73,74].

The presence of natural peptides with brominated tryptophans have made these residues a topic of considerable interest for in vitro studies. As a consequence, their synthetic production for downstream applications has also been investigated. Bittner et al. provide a comprehensive review of the synthetic methodology required to prepare brominated tryptophan, as shown in Figure 3, (**15**–**19**) [67], and bioenzymatic approaches towards halogenated aromatic amino acids have also been recently reported [75,76].

The interest in non-native amino acid building blocks is further fostered by their importance in protein research and engineering [77]. Hence, noncanonical amino acids, endowed with peculiar catalytic activity have been synthesized for introduction into peptides by solid-phase synthesis, or into proteins by mean of recombinant technologies [78,79]. With respect to the direct synthesis of halo-tryptophan, its further modification via Pd-catalysis has been reported [80], and the *L*-amino acid oxidase RebO from the actinomycete *L. aerocolonigenes* has been employed in a selective and dynamic stereo-inversion to provide the non-native *D*-halo-Trp analogues [81]. 

### 3.3. Aliphatic Halogenated Amino Acids 

**Figure 6:**
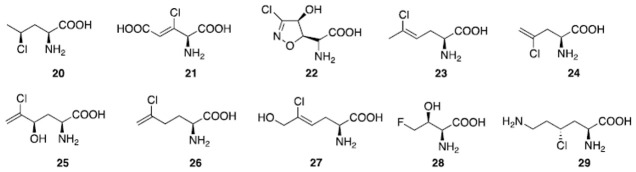
Natural aliphatic halogenated amino acids.

Aliphatic halogenated amino acids have also been described. γ-chloronorvaline (**20**, Figure 6) was identified from cultures of the microbe *Streptomyces griseosporeus* and it was found to possess antibacterial activity, especially against *Pseudomonas aeruginosa* [82]. The novel 4-amino-3-chloro-2-pentenedioic acid (**21**) was found in *Streptomyces viridogenes* [83], and is also produced by *Streptomyces xanthocidicus* [84,85]. Isoxazoline U-43,795 (**22**), displaying anticancer activity, was isolated from cultures of *Streptomyces sviceus*, and its anticancer activity was established [86]. 4-ClLys ((2*S*,4*R*)-2,6-diamino-4-chlorohexanoic acid, **29**) [5] was recently found to be an intermediate in the production of β-ethynylserine (βes), whose biosynthesis is initiated by chlorination of *L*-lysine at the C^γ^ position by the enzyme BesD halogenase. 

Several chlorinated amino acids are present in fungi [87]. For example, *Amanita solitaria*, which has a high chloride concentration, contains 2-amino-5-chloro-hex-4Z-enoic acid (**23**), and the antibacterial amino acid ((*S*)-2-amino-4-chloropent-4-enoic acid, **24**) was isolated from *Amanita pseudoporphyria* [88]. *Amanita onusta* and *Amanita miculifera* both produce ((2*S*,4*R*)-2-amino-5-chloro-4-hydroxyhex-5-enoic acid, **25**) [88] while *Amanita gymnopus* contains (**26**) [88]. The toxic "White Mushroom" (*Amanita abrupta*) provided chlorohydrin ((*S*)-2-amino-5-chlorohex-5-enoic acid, **27**) [89]. In bacteria, cultivation of *Streptomyces cattleya* in the presence of fluorine leads to the formation of 4-fluorothreonine ((*S*,Z)-2-amino-5-chloro-6-hydroxyhex-4-enoic acid, **28**) [90].

### 3.4. Halogenated Amino Acids in Non-Ribosomal Peptides

Halogenated amino acids are well represented in several non-ribosomal peptides (Table 2). These compounds are synthesized, at least in part, by one or more specialized non-ribosomal peptide synthetase enzymes, encoded by genes usually organized in a single operon in bacteria or in a gene cluster in eukaryotes [91].

### 3.5. Halogenated Amino Acids in Water and Washed Food Products in the Environment

It should be noted that free halogenated amino acids and halogenated peptides are increasingly found in drinking water and in washed foods [64]. These compounds cannot properly be considered to be native, although they are found dispersed in nature. In fact, they are formed in the environment by reaction of amino acids and peptides with disinfectants (which contain electrophilic bromine and chlorine), following a mechanism similar to that of biosynthesis. Therefore, halogenated peptides and amino acids are a class of emerging disinfection by-products (DBPs), whose toxicity and potential bioactivity are only now coming to light [64]. In this pioneering study, tyrosines were shown to be the preferred site of halogenation of peptides leading to the formation of the compounds (**1**) and (**3**)–(**10**).

### 3.6. Synthesis of Naturally Occurring and Artificial Halogenated Amino Acids

Most halogenated natural products are usually brominated or chlorinated, in a way that depends on the abundance of these elements in the environment in which they are generated. In particular, brominated products in general, including amino acids, are largely found from marine organisms, as reported in the paragraph above [99,100]. On the other hand, the biosynthesis of fluorinated natural products is relatively rare, despite the natural abundance of fluorine. The reason for this could be due to both its high electronegativity and the high enthalpic cost associated with the fluorine desolvation [101]. The introduction of polar C-halogen bonds deeply impacts on the potential bioactivity of natural organic compounds. Fluorination makes no exception and a significant effect has often been reported on introduction of fluorine into bio-organic compounds. The native introduction of fluorine by enzymatic processes is still not well understood, although some enzymatic reactions have been described for some bacteria, such as *Streptomyces cattleya*, and it is used in chemoenzymatic synthesis [102,103]. There is an ongoing interest in understanding the mechanism by which halogenation occurs. This may help to design or optimize strategies to synthesize halogenated amino acids. For example, several studies have focused on the use of fluoro-pyruvate as a substrate for type II aldolases, to synthesize some chiral organo-fluorines [103]. The demand of halogenated amino acids led to development of several artificial strategies to provide these compounds for further rational design of more complex molecules. 

The synthesis of halogenated amino acids has already been reviewed and the excellent chapter by Strickland and Willis remains an important reference [104], as well as the methods of preparation of brominated tryptophan (**15**–**19**) reported by Bittner et al. [67]. The fermentative production of the halogenated amino acid 7-chloro-L-tryptophan from sugars, ammonium and chloride salts has also been achieved. This required metabolic engineering of an L-tryptophan producing *C. glutamicum* strain for expression of the genes coding for FAD-dependent halogenase RebH and NADH-dependent flavin reductase RebF from *Lechevalieria aerocolonigenes* [105]. 

**Scheme 1:**
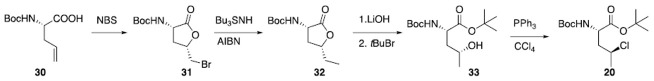
Synthesis of γ-chloronorvaline (**20**).

A preparation for γ-chloronorvaline (**20**) has been recently reported (Scheme 1). Starting from amino acid (**30**), bromoetherification carried out using N-bromosuccinamide provided bromo lactone (**31**). This was dibrominated using an established radical procedure. Resulting ester (**32**) was subjected to hydrolysis and esterification to give alcohol **33** which was finally converted to desired (**20**) via Appel reaction [106]. 

**Scheme 2:**
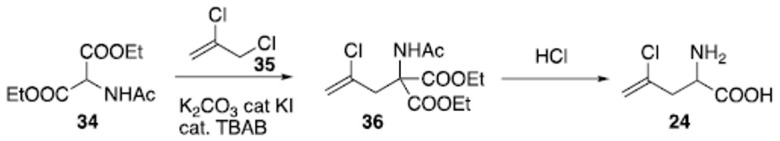
Synthesis of racemic (S)-2-amino-4-chloropent-4-enoic acid (**24**).

The racemic preparation of amino acid (**24**) has been recently reported on a large scale (Scheme 2). Starting from acidic aminomalonate derivative (**34**), its reaction with alkyl chloride (**35**) under phase transfer catalysis [107,108,109,110,111,112,113,114] operated by tetrabutylammonium bromide (TBAB) in the presence of catalytic potassium iodide, generated adduct (**36**). Hydrolysis and acidic condition promoted decarboxylation and provided compound (**24**) as a racemate. 

**Scheme 3:**
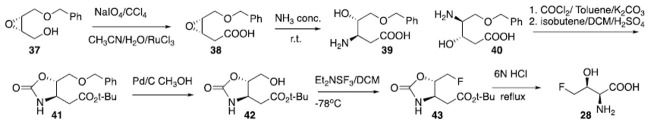
Preparation of 4-fluorothreonine (**28**).

Many biosynthetic pathways relying on the reaction of fluoroacetaldehyde and glycine under the catalysis of threonine transaldolases have been reported for producing 4-fluorothreonine (**28**) [115]. A synthesis has been published by Scolastico (Scheme 3) [116] starting from epoxide (**37**). Oxidation of the alcohol using periodate as the terminal oxidant under the catalysis of Ru(III) provided carboxylic acid (**38**) which was then reacted with concentrated ammonia to give regioisomers (**39**) and (**40**) in a 9:1 ratio. Following reaction of **39/40** as a mixture with phosgene and isobutene produced the desired protected amino acid (**41**), after chromatographic purification. Subsequent debenzylation using Pd/C and methanol provided compound (**42**), which was then used as the starter for the electrophilic deoxyfluorination to give carbamate-protected 4-fluorotheonine (**43**). Final deprotection to (**28**) was achieved via treatment with 6N HCl [116]. Potenti et al. recently reported another synthetic route avoiding the use of Ru(III) [117]. The preparation of 4-chloro-lysine (**29**) has been reported by reacting commercially available lysine hydrochloride with Cl_2_ under irradiation with UV light. The reaction provided the halogenated amino acid (**29)** in 11% isolated yield [118]. Fluoro-amino acids are pivotal to many medicinal and agricultural chemistry applications and represent one of the more promising and rapidly developing areas of research in organic, bio-organic and medicinal chemistry [119]. Their synthetic pathways and the fluorinated versions of alkyl, cyclic, aromatic amino acids were recently exhaustively reviewed [120] and several examples and applications have been reported in another review [119]. Moreover, the synthetic methods to access to amino acids containing multiple fluorine atoms such as CF3, SF5 and perfluoroaromatic groups were also recently reported [121]. 

## 4. Halogenated AMPs

### 4.1. AMPs as Novel Drug Candidates and Their Mechanism of Action 

Antimicrobial peptides (AMPs) are compounds that can be potentially used as alternatives to conventional antibiotics [122] or effective adjuvants that can help conventional antibiotics to overcome resistance [123,124,125,126,127]. However, AMPs still have critical issues that prevent them from clinical application and require further optimization. Therefore, these compounds represent an intriguing opportunity to observe how halogenated amino acids may tune some physico-chemical properties of AMPs that are responsible of various aspects of their biological activity.

Endogenous AMPs, often referred to also as host-defense peptides (HDPs) [128], are ancient, gene encoded innate immune effectors. They are ubiquitously found in both prokaryotic and eukaryotic organisms and display a remarkable level of structural and functional diversity. In prokaryotes, they play a role in competition for resources with other strains, in nutrient-poor environments. In eukaryotes they have a fundamental role in keeping commensal organisms under control, and as a first line of defense against microbial infections [129,130]. 

In addition to a direct antimicrobial activity, many AMPs also exert immunomodulatory or healing-promoting activities, making them interesting molecules for the development of novel therapeutics [131,132]. 

AMPs are classified into several categories based on common structural features and/or conserved sequence motifs.

(a) the members of the *α family* of AMPs have disordered and flexible structure in solution but adopt an amphipathic helical structure in the membrane environment [133]. It includes anuran, magainins and temporins, insect cecropins and some mammalian cathelicidins [134].

(b) the members of the *β* *family* of AMPs have two or more β-strands that adopt β-hairpin or β-sheet structures in aqueous environment and are internally stabilized by one or more disulfide bonds [135,136], that are further stabilized by contact with a lipid surface. They are more rigid than the members of the α family peptides, and include invertebrate tachyplesins and thanatin, and the mammalian lactoferricin B and the cathelicidins protegrin and bactenecin-1 (β-hairpins) [137], as well as mammalian α-defensins (β-sheets) [138,139].

(c) the members of the *α/β family* of AMPs have both α−helical and β−sheet structures, such as fungal and insect defensins and mammalian β-defensins [139]. Another example is the plant-derived circulin A characterized by a combined α-helix and β-sheet structure based on cyclic cysteine knot [140].

(d) the members of the *non-α/β* or *loop family* of AMPs, are characterized by peptides that do not have α-helical or β-strand content but rather have loop-shaped structure that can be stabilized by disulfide, amide or isopeptide bonds [141]. These include post-translationally modified prokaryotic peptides such as the lantibiotics nisin and mersacidin (lanthanide bridged) [91,142,143]. This family also includes linear sequences classified according to the presence of a prevalent amino acid present in their sequence, such as glycine-, serine-, tryptophan-, arginine- and proline- or histidine-rich peptides [144]. 

**Figure 7:**
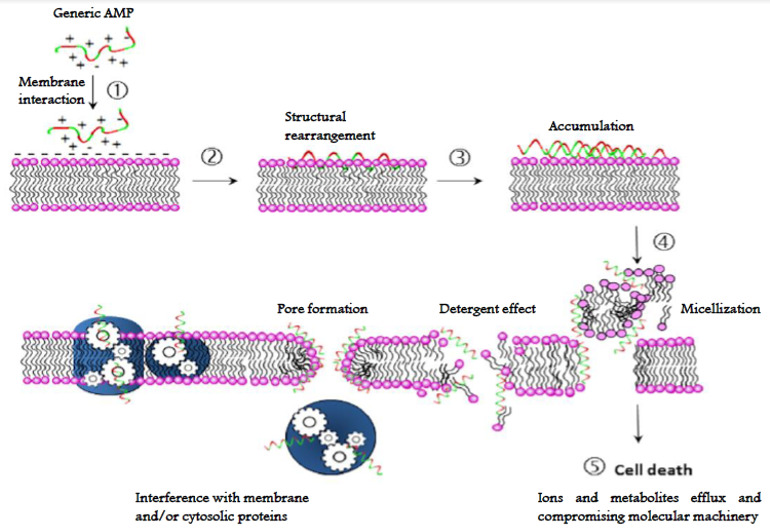
Generalized mode of action of AMPs. (1) Electrostatic and hydrophobic interaction with cell surface; (2) conformational rearrangement and membrane insertion; (3) accumulation to a threshold level (4) membrane alteration (pore or lesion formation, detergent effect, micellization, etc.) leading to permeabilization and/or depolarization. Membrane or cytoplasmic protein machinery may also be disrupted; (5) inactivation of the microorganism.

The mechanism of action of AMPs, unlike most of the conventional antibiotics, is not usually based on a specific molecular target, since AMPs interact directly with microbial membranes on which they exert a destabilizing and damaging effect known as “lytic” [145,146,147]. Their structural properties (amphipathicity, cationic charge, shape and size) allow them to interact with and efficiently permeabilize [148] the microbial lipid bilayer. Interaction with the target cell membrane can be described by five distinct steps: (1) contact with cell surface of target cells based on physical affinity (electrostatic and/or hydrophobic interactions); (2) conformational rearrangement at the membrane surface and membrane insertion; (3) accumulation up to a threshold level (4) an alteration of the membrane occurs by either permeabilization or depolarization or causing any other membrane misfunction (detergent effect, micellization, etc.) through a direct or indirect abnormality (this step may involve peptide oligomerization); (5) killing of the microorganisms through a lytic process of the membranes or any other irreversible processes within the cell [91] (Figure 7). 

Most of AMPs act by forming discrete or toroidal pores. These peptides assemble on the membrane surface among the phospholipid head groups and promote disruption of bilayer curvature in a detergent-like manner, causing the membrane disintegration and, consequently the cell death [149]. Although membrane permeabilization is the most frequent mechanism of action of AMPs, some of them can also penetrate into the microbial cytoplasm and then inhibit vital intracellular processes such as protein synthesis, nucleic acid synthesis, or specific enzymatic activities (Figure 7) [150,151,152,153].

As reported by Tossi et al. [134,154,155], some physico-chemical features are important to determine the structure-activity relationship of AMPs, such as a net positive charge (which generally ranges from +4 to +6 but can have higher values), which is crucial to promote the interaction with the anionic surface of microbial membranes and the subsequent interaction-mediated lysis mechanisms [136]. For this reason, the antimicrobial efficacy of AMPs in killing bacteria is better expressed at a lower pH, at which all cationic residues are protonated [156] and at low salt concentration, especially divalent cations, allowing these peptides to better interact with the bacterial surface. The hydrophobicity of AMPs instead determines their capability to penetrate into lipid bilayers [136] so that the ratio hydrophobic to charged residues is an essential determinant parameter to design AMPs [157]. The ability to adopt an amphipathic structure is particularly important for interaction with membranes to generate pores or lesions, so that there is an approximate equivalence between the number of polar and hydrophobic residues in most AMPs [133,134]. The secondary structure is a fundamental feature of AMPs, with helical or β-sheet stretches adopted or arranged for optimal insertion into the membrane bilayer. As a consequence of this generalized disruption and lack of specific targets, bacteria have difficulty to develop permanent resistance against AMPs. However, the use of this new class of antibiotics is mainly limited by in vivo toxicity, as they can also interact with (and damage) animal cell membranes to some extent. Other problems are related to the peptidic nature of these molecules, including poor bioavailability due to susceptibility to proteases and rapid clearance, potential antigenicity, and the high cost of production. To address some of these limitations, some strategies involve incorporation of non-proteinogenic amino acids [158,159], which are useful tools for modulating essential AMP-like properties based on native AMPs. They can be used to optimize size, backbone stability and/or conformation, side-chain properties such as hydrophobicity or charge, and overall properties such as amphipathicity. Other strategies are based on the design of hybrid peptides [160,161,162] to discover innovative drug leads with high cell selectivity and/or improved bioavailability [163,164]. 

### 4.2. Ribosomally-Synthesized Halogenated AMPs 

In this paragraph only gene-encoded, ribosomally synthesized halogenated AMPs will be considered (Table 3). Halogenation, in the specific case bromination, is introduced as a post-translational modification at the level of tryptophan, and to our knowledge always in position 6 of the indole ring, at least in all the cases where the site of substitution has been investigated. In addition, all these halogenated peptides have been isolated from marine organisms, likely as a consequence of the relative abundance of bromide ion in sea water (0.9 mM) and of the expression of more or less specific (halo)peroxidases that can catalyze the bromination reaction in these organisms.

The first AMPs with Br-Trp in their sequence were isolated from intestinal tissue of the Atlantic hagfish (*Myxine glutinosa*), a jawless primitive chordate whose defense from pathogenic microorganisms appears to depend solely on innate mechanisms, as it lacks components of the adaptive immune system such as circulating lymphocytes and antibodies, the production of which evolved only in the jawed fish [171].

In particular, three AMPs with a high content of in cationic residues were isolated from *Myxine glutinosa*, which were named Hagfish Intestinal Antimicrobial Peptides (HFIAP-1, -2 and -3, see Table 3). These were chemically sequenced and HFIAP-1 and -2 found to have identical amino acid sequence of 37 residues, differing only for the presence of two Br-Trp in HFIAP-1 (W7 and W32) and one in HFIAP-2 (W7). HFIAP-3, was shorter (29 residues), although its sequence is quite similar to that of the other HFIAP, and had brominated W7. The non-brominated synthetic peptides HFIAP-1 and -3 were tested on a wide panel of Gram-(+) and Gram-(-) bacteria and in most cases showed broad-spectrum antibacterial activity in the concentration range of 2-16 μg/ml. It was also shown that the presence of bromine is not critical for the antimicrobial activity, but appears to protect the peptides from the hydrolytic activity of proteases [165,171]. 

Cloning of HFIAPs resulted in two cDNA sequences that, surprisingly showed that these peptides were derived from prepropeptide precursors of the cathelicidin family [165]. They were the first non-mammalian cathelicidins to be found and confirmed this to be an ancient family of AMP precursors that is now believed to be present in most vertebrates, ranging from proto-chordates to jawed fish, anurans, reptiles, birds and placental and non-placental mammals [172]. Furthermore, it is uncertain how the bromination is carried out at the tryptophan level, an unusual modification for vertebrate peptides. Haloperoxidases are responsible for halogenation of ring systems in a wide variety of marine invertebrates but it is not known if endogenous peroxidases are also acting in the hagfish, and what effect bromination has on peptide.

Styelins are Phe-rich, linear α-helical peptides isolated from the tunicate *Styela clava*, which are extensively post-translationally modified and that display antimicrobial activity against a wide panel of pathogens. Cloning of the cDNA of the three identified styelins showed these peptides to be synthesized as precursors with a 22 residues signal sequence followed by the 31 or 32 residues mature peptide (plus a glycine residue acting as a C-terminal amidation signal for the mature AMP) and then a C-terminal pro-piece of 26 residues [173]. Styelin D, a 32 residue, C-terminally amidated peptide, has been extensively investigated and found to contain 12 residues that are post-translationally modified. These included 6-bromotryptophan at position 2, in addition to one dihydroxyarginine, four 3,4 dihydroxyphenylalanines, two 5-hydroxylysines and four dihydroxylysines. These modifications improve the bactericidal activity of styelin D at high salt concentration and acidic pH, but it is not clear whether bromination contributes to this activity [166].

Hedistin is a 22-residue amidated peptide isolated from the celomocytes of *Nereis diversicolor*, a marine annelid [167]. The native peptide contains Br-Trp at positions 4 and 18 of the sequence, likely at position 6 of the indole ring, and is constitutively expressed as a pre-propeptide precursor that is processed before release. The antimicrobial activity was tested with both the native brominated peptide and a synthetic peptide without bromine. Both forms displayed an equivalent activity against a small panel of reference bacteria, indicating that bromination is not essential for antibacterial activity. The synthetic form was then tested against a larger panel of Gram-(+) and Gram-(-) species and was active against most of the former strains, including Methicillin-Resistant *Staphylococcus aureus* (MRSA), while among Gram-(-) species only *Vibrio alginolyticus*, a marine bacterium that causes episodes of mass mortality of larvae of bivalves of commercial interest, was susceptible [167].

AMPs containing brominated tryptophan have also been found in sea urchin species, the first being identified in the green sea urchin *Strongylocentrotus droebachiensis* and named strongylocin 2. This 51 or 52 residue peptide has the N-terminal Trp is brominated [168], and it has six Cys residues forming three disulfide bonds. It is synthesized as pre-propeptide of 89 or 90 residues, depending on the isoform, and the mature peptide isolated from coelomocytes, was tested against both Gram-(+) and Gram-(-) bacteria and displayed a potent antibacterial activity with MIC values below 5 μΜ and was not hemolytic at a fourfold higher concentration [168].

Centrocins 1 and 2 were also later isolated from coelomocytes of *S. droebachiensis* [170], and these peptides have a heterodimeric structure consisting of heavy (30 residue) and light (12 residue) chains that are linked via an intermolecular disulfide bond. The Trp present at position 2 of both centrocins is brominated as indicated by MS measurements [170]. Native centrocins display an antibacterial activity that is comparable to that of strongylocins, and which is due to the heavy chain, which can adopt an amphipathic α-helical conformation, as suggested by helical wheel projections, while the light chain alone was inactive. A synthetic version of the heavy chain lacking bromine demonstrated that bromination is not required for antimicrobial activity. 

BLAST searches have identified putative polypeptides showing similarity to strongylocins and centrocins in the genome data-base of the purple sea urchin *S. purpuratus*. Finally, two centrocins (EeCentrocins 1 and 2) and a strongylocin (EeStrongylotin 2), that are homologous to those isolated from *S. droebachiensis*, were isolated from the hemocytes of the edible sea urchin *Echinus esculentus* [169]. All the mature peptides contain Br-Trp (Trp2 and Trp3 in EeCentrocin 1, Trp1 and Trp9 in EeCentrocin 2 and Trp1 in EeStrongylotin 2). NMR spectroscopy confirmed that the bromine is in position 6 of the indole ring. As for the antibacterial activity, the results are similar to those of the homologous peptides from *Strongylocentrotus droebachienshis*, i.e., the peptides are active both against Gram-(+) and Gram-(-) strains. The heavy chain is responsible for the antibacterial activity, and this seems to be independent of Trp bromination. EeCentrocins 1 and 2 proved moderately active also against some fungi and had only a limited hemolytic activity up to a concentration of 100 μM [169].

An interesting case of AMP halogenation is the lantibiotic NAI-107, a 24-residue peptide produced by genera *Microbispora* and *Actinoallomurus* [174,175], which is active in experimental models of infections [176,177]. Lantibiotics are ribosomally synthesized peptides that are post-translationally modified and are characterized by a variable number of the thioether amino acids lanthionine or methyl-lanthionine, which form intramolecular rings [178]. Most of the lantibiotics are effective against Gram-(+) bacteria and act by binding to the lipid II, a key intermediate in peptidoglycan biosynthesis, thereby inhibiting the assembly of the cell wall [179]. NAI-107 is characterized by the presence of two unusual amino acids, namely 5-chlorotryptophan and 3,4 dihydroxyproline [174]. The lantibiotic is produced as a complex of congener peptides differing in the extent of Pro14 hydroxylation with no, one or two hydroxy groups (respectively A0, A1 and A2 congeners), and for the presence or absence of chlorine on Trp4 (F congeners) [180]. Antimicrobial activity assays showed that the three A congeners displayed comparable activity with A0 being slightly less active than A1 andA2. Conversely, the congeners lacking the chlorine atom at Trp4 were less active that those with the halogen atom. This represents therefore a difference with respect to what was observed with Trp bromination in eukaryotic AMPs as described above, in which the presence or absence of the halogen has no effect on antimicrobial activity. The increased activity in Cl-containing NAI-107 F-congeners has been attributed to the increased hydrophobicity caused by the chlorine on Trp4 that might improve the interaction of the peptide with lipid II [180].

Brominated variants of NAI-107, named NAI-108, were obtained by growing a producer strain of *Actinoallomurus* in a medium in which NaCl was substituted by KBr at the same concentration (8.6 mM) [175]. In these variants, the single chlorine atom in Trp4 was replaced by bromine. The antibacterial activity of brominated congeners was compared to that of chlorinated equivalent congeners, and interestingly, this activity was in general improved by 1-2 MIC values when tested against a panel of Gram-(+) human pathogens that included multi-resistant strains such as MRSA and vancomycin-resistant *Enterococcus faecalis* [175].

### 4.3. Synthetic Halogenated AMPs

Native AMPs and their synthetic analogues have received increasing attention from academic research and the pharmaceutical industry [181]. However, despite their promising antibiotic properties, they suffer from several disadvantages as peptide therapeutics, for example the susceptibility to proteolytic degradation, the pH- and/or salinity-dependence of their activity, and loss of antimicrobial effect due to binding to serum components [182,183], as well as a potential toxicity to mammalian cells. Moreover, the costs of industrial production are high. For these reasons, modifications to AMPs have been developed with the aim to improve antimicrobial potency and stability while at the same time to reduce toxicity and size. This can be done by chemical modifications such as substitution of one or more amino acid residues of the natural AMP template with other L- or D-amino acids, both proteinogenic or non-proteinogenic, as well as N-terminal acetylation, C-terminal amidation, peptide cyclization (usually via disulfide bond formation and more rarely by backbone cyclization), or introduction of unnatural amino acids to synthesize α-peptoids [184], β-peptoids [185], β-peptides [186], peptide/peptoid hybrids [187,188,189] and/or functionalized with lipo-amino acids [190] or with PEG [191]. 

Fluoro amino acids are introduced into peptides or proteins as internal probes to study inter and intramolecular interactions, as well as for structural stability and dynamics measurements [192,193,194]. The fluoro amino acids are a useful tool to explore their mechanisms of action as the Fluorine-19 nucleus constitutes an excellent NMR probe due to a large chemical shift dispersion, high sensitivity to its local environment, and low background noise caused by the absence of fluorine in biological molecules [195,196]. 

Fluorination is also quite a common substitution in medicinal chemistry to alter pharmacological properties. It has also been applied to AMPs, e.g., to prepare highly fluorinated analogues of magainin [197,198], and melittin using 5,5,5-trifluoroleucine [199] or in the synthesis of peptidic libraries of buforin and magainin, obtaining analogues with enhanced antimicrobial activity, increased resistance to proteolytic degradation and decreased hemolytic activity [200]. The introduction of fluorine, chlorine, bromine and iodine atoms into the honeybee peptide jelleine-1 [201] via halogen-substituted phenylalanine also led to improved protease stability. Interestingly, a fluorinated analogue showed antimicrobial activity similar to that of the parent peptide, while chlorinated, brominated and iodinated analogues displayed a two to eightfold increase in their in vitro activity. However, although the iodine analogue displayed the highest activity in vitro, the chlorinated and brominated analogues displayed a greatest potency in vivo [201]. 

A recent study [202] on tritrpticin and its amidated analog tritrp1, which are AMP members of the Trp/Arg-rich family with a strong antimicrobial activity against bacteria and fungi [203], showed that fluorination preserved the antimicrobial activity as well as the mechanism of action. The authors adopted a recombinant approach for the production of fluoro tritrpticin analogues containing F-Phe, F-Tyr and F-Trp and studied the effect both on the expression of peptides and on their activity. Analogues containing all three F-Trp exhibited a strong concentration-dependent permeabilizing activity against negatively charged membranes similarly to native tritrpticin and tritrp1 [204]. F-Trps are attractive candidates because of their slightly higher hydrophobicity [205,206], while maintaining the size of proteinogenic Trp. Interestingly, the change in the dipole moments [193,207] for the F-Trp did not affect the antimicrobial activity of these peptides, while a decrease of activity was observed when F-Tyr was introduced, likely due to the effect of fluorination on the acidity of the -OH group as well as on the overall hydrophobicity [202].

A peptidic mimetic library of the antimicrobial battacin, containing fluorinated aromatic L-serine derivatives in place of a key D-phenylalanine residue, has been reported [208]. These linear cationic lipopentapeptides, derived from the octapeptin battacin [209], were modified by substituting the original D-Phe residue with FPhe and modified L-phenylserine residues, obtained via Mitsunobu reactions of phenols with the serine OH group [210]. This modification enhanced the broad-spectrum inhibitory activity against a wide array of pathogenic species including clinically relevant bacteria such as *P. aeruginosa* and *Campylobacter jejuni*.

With respect to α-peptide/β-peptoid hybrids, in recent studies, the influence of hydrophobicity, fluorination, and distribution of cationic/hydrophobic residues on antimicrobial, hemolytic, and cytotoxic properties were investigated [211,212]. The authors first investigated the importance of distance between the amino functionalities and the backbone for the antimicrobial properties. No general trend was observed for potency against Gram-(-) bacteria with respect to structural variations, while a modest increase in the antimicrobial activity against *S. aureus* was observed when shorter cationic side-chains were introduced, likely dependent on the increased hydrophobicity. They then studied the effect of the hydrophobicity of the aryl side-chain moieties variously modified by methylation, fluorination and trifluoromethylation at the 4-position of the phenyl rings of the β-peptoid. Fluorination resulted in a significant increase in activity against *S. aureus* and *E. faecium*, while the activity remained unchanged against *P. aeruginosa*, *E. coli*, *Acinetobacter baumannii* and *Salmonella typhimurium* [211].

Finally, the hemolytic activity was investigated as an indicator of cytotoxicity. Limited fluorination at a single position in the aromatic β-peptoid residues significantly increased activity against both Gram-(+) and Gram-(-) bacteria without affecting hemolysis [211,212]. By contrast, trifluorination or methylation of all rings led to a decreased selectivity between bacterial and red blood cells [211]. The introduction of halogenated hydrophobic end groups (such as 4-Cl phenyl-acetic; 5-Br indol-acetic; pentafluoro-phenyl acetic; etc.) increased the activity against *S. aureus, E. coli* and *P. aeruginosa*, although this also resulted in a slightly reduced cell selectivity [212]. 

Poly-*N*-substituted glycines (α-peptoids) are a class of peptidomimetics in which the side chains are moved to the backbone amide nitrogen [213]. In 2003, the first example of antimicrobial peptoids [214] was developed and since then several others have been found to efficiently retain antimicrobial activity, while improving proteolytic stability and bioavailability with respect to the corresponding natural peptides [158,215]. Some fluorine substituted peptoids had enhanced antimicrobial activity against both Gram-(+) and Gram-(-) bacteria without toxicity to mammalian cells [216]. Recently, a study on the effect of the halogenation in α-peptoids investigated the relationship between the nature of the halogen and the degree of halogenation, their ability to self-assemble into nanostructures and their antimicrobial activity [217]. Sets of scaffolds of four sizes were used containing alternating NLys and NPhe units, where F, Cl, Br and I atoms were introduced into position 4 of the phenyl rings. Each set contained a non-substituted control, four fully halogenated peptoids and four “half substituted” peptoids, in which every second phenyl ring was substituted with a halogen atom. In general, a correlation was observed between the antimicrobial activity against Gram-(+) strains, and the nature of the halogen (Br > Cl > F) and degree of substitution, linked both to an increase in hydrophobicity and altered self-assembly properties of the compounds. However, the activity of peptoids started decreasing above a certain level of hydrophobicity. The brominated α-peptoids showed a >30-fold increase in activity against *S. aureus* and 16- to 64-fold increase against *E. coli* and *P. aeruginosa*, without any increase in cytotoxicity [217]. 

**Figure 8:**
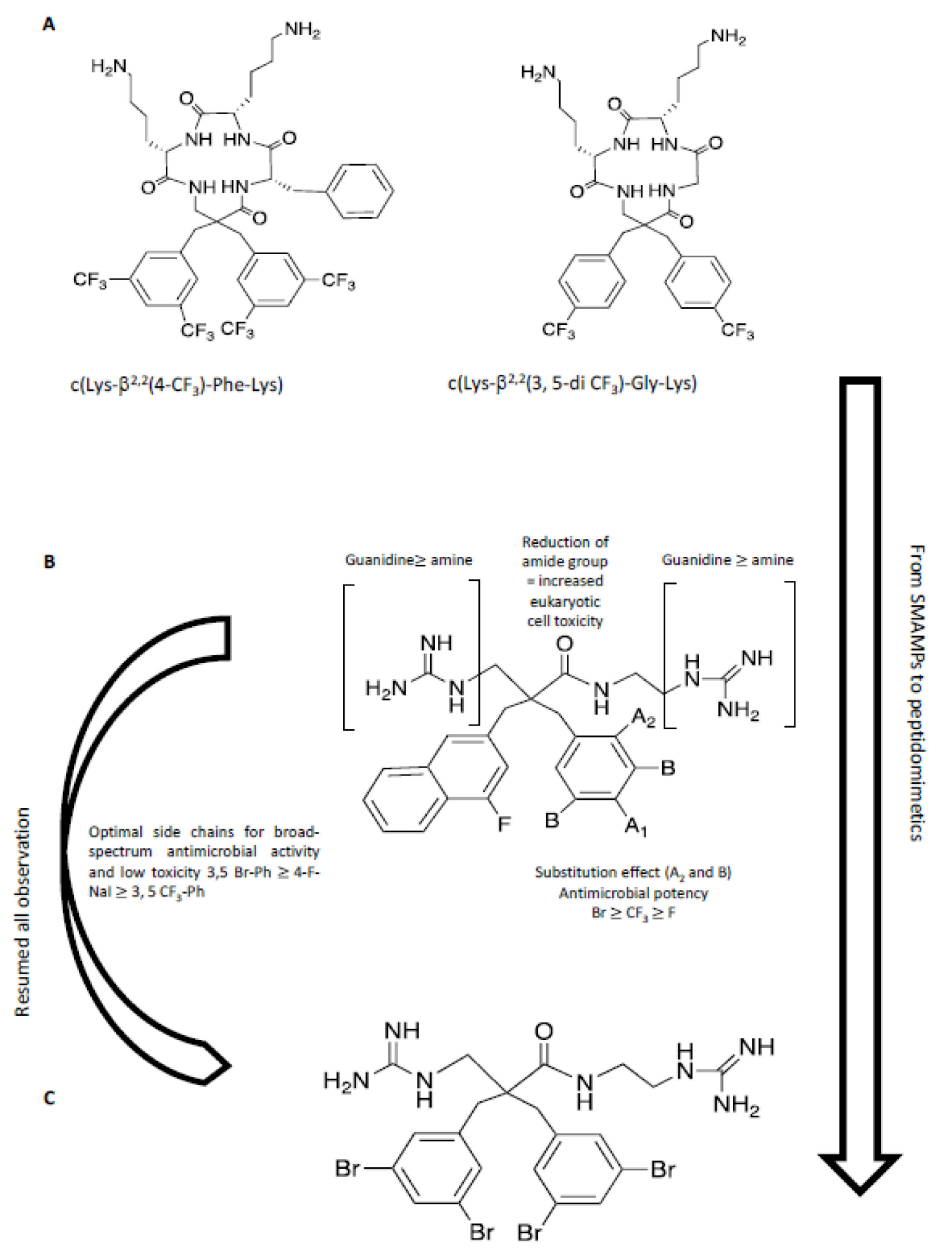
In this figure, a hierarchical approach from peptide to peptidomimetic is resumed. (**A**) From SMAMPs [219]: a peptide scaffold was used to investigate the effect of halogenation and hydrophobicity. (**B**) Summary of SAR for peptidomimetics [220]. (**C**) The optimized molecule with high antimicrobial activity and low toxicity in preliminary test in vitro and *in vivo* [220].

A recent study has described the incorporation of two halogenated α,α-disubstituted β^2,2^-amino acid residues [218] into an amphipathic cyclic tetrapeptide [219] (Figure 8) with general sequence c(Lys-β2,2-Xaa-Lys), where Xaa = Lys, Gly, Ala, or Phe. Antimicrobial activity was in the low μg/mL concentration range against multi-resistant Gram-(+) and Gram-(-) strains, with little toxicity against human red blood cells, in particular for the halogenated analogues c(Lys-β^2,2^(4-CF_3_)-Phe-Lys) and c(Lys-β^2,2^(3,5-di-CF_3_)-Gly-Lys). These two peptides were tested against 30 multi-resistant clinical isolates, maintaining the antimicrobial activity. A library of synthetic mimics of antimicrobial peptides (SMAMPs) was prepared, in which the peptide features were minimized [220] to optimize both antimicrobial potency, pharmacokinetic properties, and to reduce toxicity. The library of SMAMPs containing halogenated α,α-disubstituted β-amino amides, amines and guanidines had high antimicrobial activity, which was in general improved by increasing the net positive charge (guanidine > amine). In particular, di-guanidine derivatives containing halogen substituents showed broad-spectrum antimicrobial activity. 3,5-Br-benzylic derivates were especially potent against several clinical pathogens isolates in hospitals, such as *S. aureus* and *E. faecium* with minimum inhibitory concentrations (MIC) of 2-4 μg/mL. This mimic also displayed activity against multi-resistant Gram-(-) bacteria, i.e. *E. coli* and *P. aeruginosa*, with MIC values of 4-8 μg/mL. Moreover, the results showed that the most potent 3,5-Br-benzylic derivates had MIC values close to oxytetracycline, used as reference antibiotic. Halogenated lipophilic groups, 3,5-Br-Phenyl, 3,5-CF_3_-Phenyl and 4-F-1-Naphtyl, enhanced antimicrobial potency, without modifying the toxicity towards eukaryotic cells and, at the same time, improving the stability in vivo.

## 5. Future Perspectives and Conclusions

This review has been focused on the structural and biological effects of introducing halogen atoms into amino acid residues. Special emphasis was placed to natural and synthetic peptides with antimicrobial activity. Primarily, we showed that halogenation can affect the structure, and in particular the conformations that can be adopted by peptides as well as protein folding. Because of particular physico-chemical properties of halogens, their use in modified amino acids therefore becomes a powerful tool to study the mechanisms and dynamics of these processes. Furthermore, halogenation, such tryptophan bromination, resulted to be useful to optimize [221] the characteristics of bioactive molecules with pharmaceutical activity, as shown for products from marine organisms. Fluorine is efficaciously used as an internal probe for F^19^-NMR studies of peptide and protein structure-activity relationships (SAR), especially in interaction with biological membranes [18,222]. SAR studies have also revealed a correlation between the enhanced hydrophobicity resulting from halogenation and the antimicrobial activity of AMPs and their pep-tidomimetics. This could help to design such molecules with a finely tuned balance between the charge and hydrophobic features to optimize the pharmacological profile [223,224]. The synthesis of SMAMPs requires the application of several skills such as organic synthesis, structural and conformational studies and appropriate testing to better understand the biological features at the basis of potency, stability, bioavailability and selectivity, and assist in the rational design of new molecules [217]. It has been recently shown that halogenated amino acids are also tools for other applications in pharmaceutical and organic chemistry. Bromotryptophan can be in fact utilized as a scaffold for the synthesis of new organic compounds [225]. Different types of halogenated Trp, prepared by employing specific halogenases (e.g., marine haloperoxidases) or haloindoles during biosynthesis by tryptophan synthase, can be introduced into peptides or proteins to tune biocompatibility and activity [226]. Moreover, the use of halogenated Tyr has been recently shown to increase the binding affinity of an antibody towards its antigen [14].

In conclusion, this review aims to highlight how biological and chemical methodologies can interact very constructively in the study of bioactive peptides. An example is the recent developments of halogenated peptidomimetics built from natural AMP templates. The present work would like to address both the biologist and the chemist who begin to approach AMPs, and inform them of the potential of peptide and protein halogenation, in a spirit of multi-disciplinarity that is typical of both the academic and research activities of prof. V. Hruby.

## Figures and Tables

**Table 1:** Kinetic refolding parameters of Trx1P, Trx1-(4R)-FPro, Trx1-(4S)-FPro, and Trx1-(4,4)-FPro.

	*k of I_trans_→N_cis_ [s^−1^]*^a^ (In the Context of Tertiary Structure)	Normalised Rate for *k of I_trans_→N_cis_*	Ref.
**Trx1P**	9.3 ± 0.5 × 10^−5^	1	[35]
**Trx1-(4*R*)-FPro**	8.7 ± 2.5 × 10^−5^	0.94	[27]
**Trx1-(4*S*)-FPro**	8.0 ± 2.5 × 10^−4^	8.6	[27]
**Trx1-(4,4)-FPro**	7.9 ± 0.7 × 10^−5^	0.85	[35]

^a^ Values determined by N-tests.

**Table 2:** List of a selection of halogenated non-ribosomal peptides, adapted from Ref. [92].

Name	Structure	Halogenated Amino Acid	Source	Ref.
**Orbiculamide A**	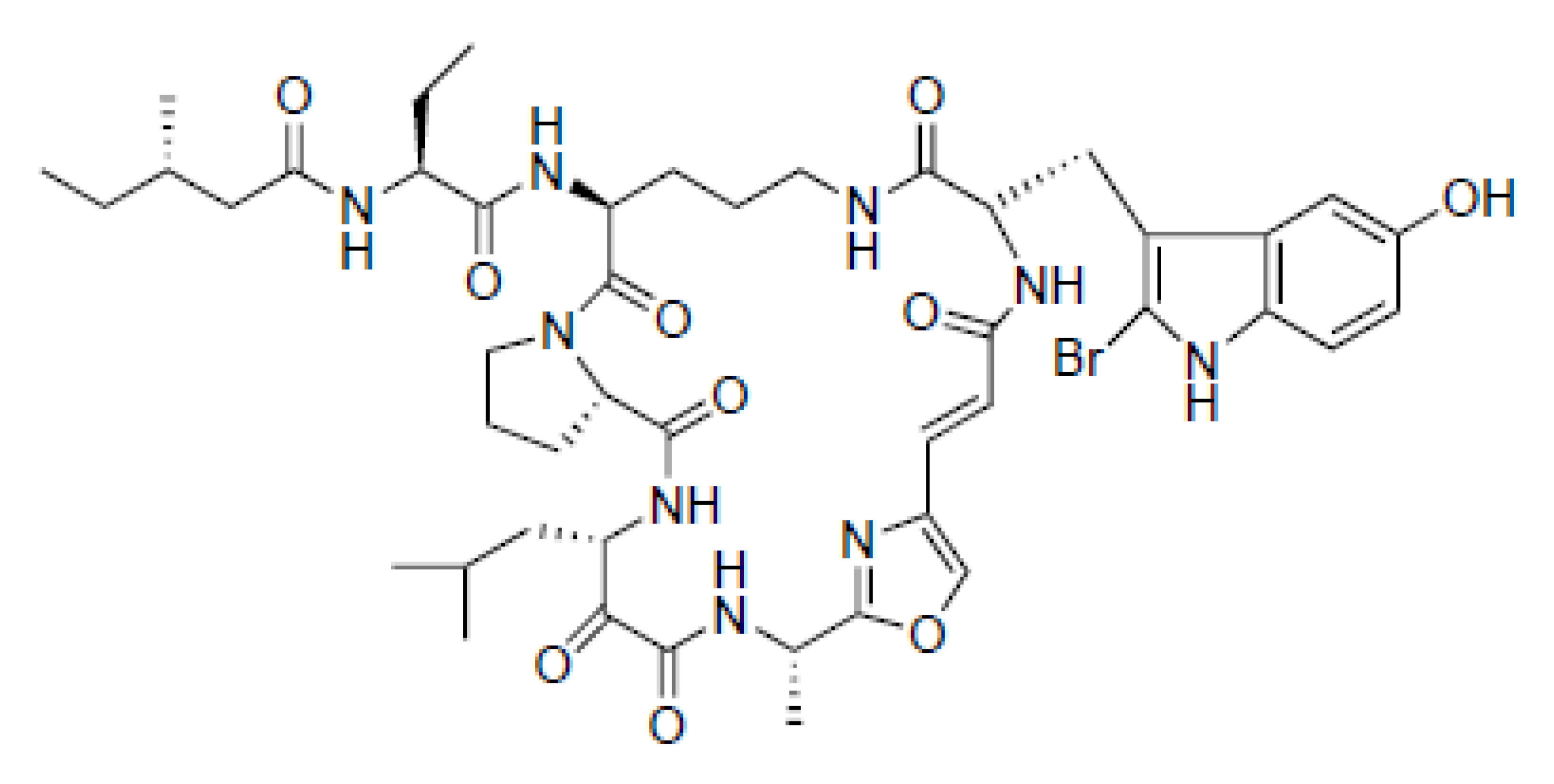	2-Bromo-5-hydroxytryptophan	*Theonella* spp.	[68]
**Keramide H**	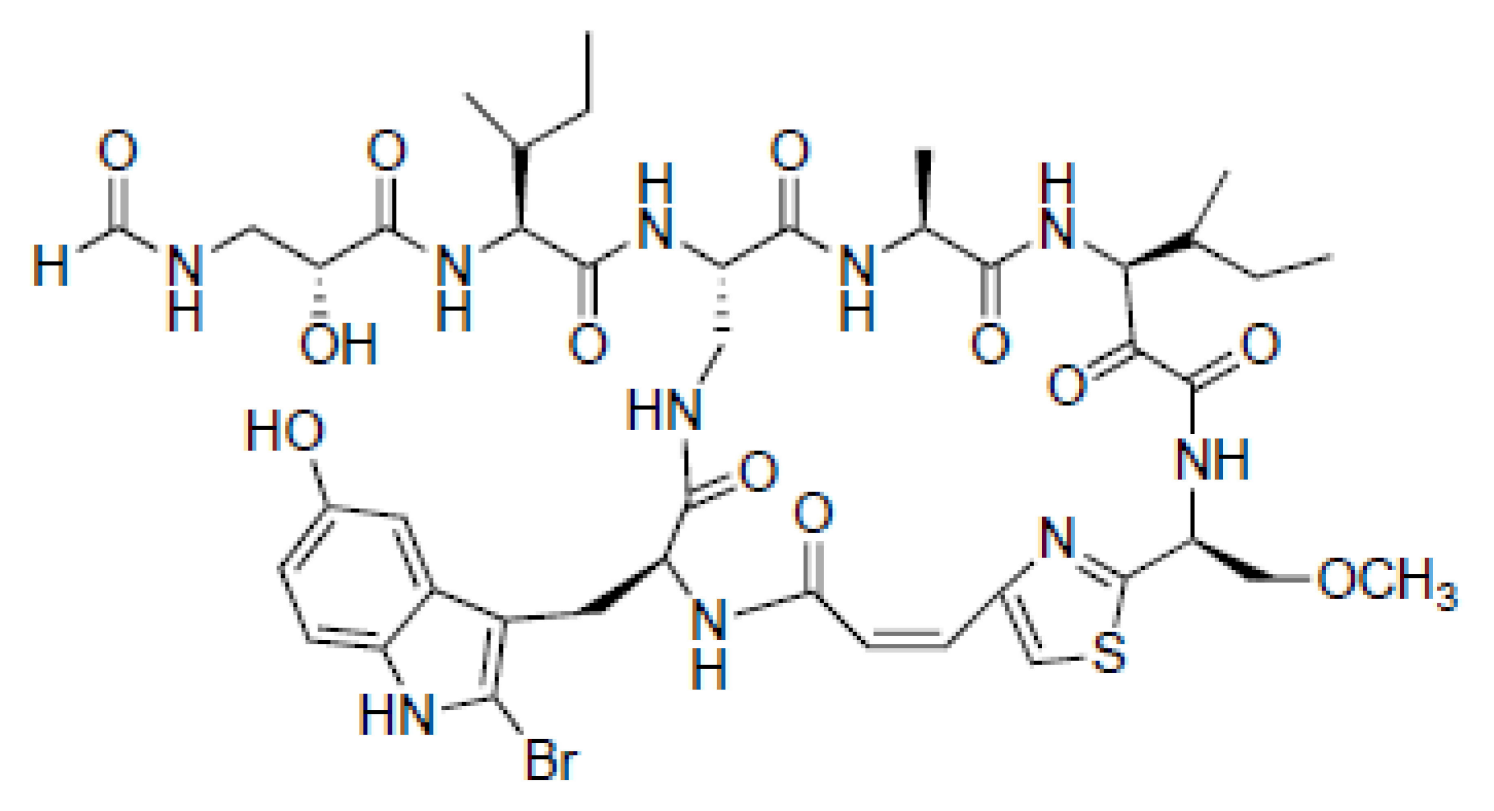	2-Bromo-5-hydroxytryptophan	*Theonella* spp.	[69]
**Konbamide**	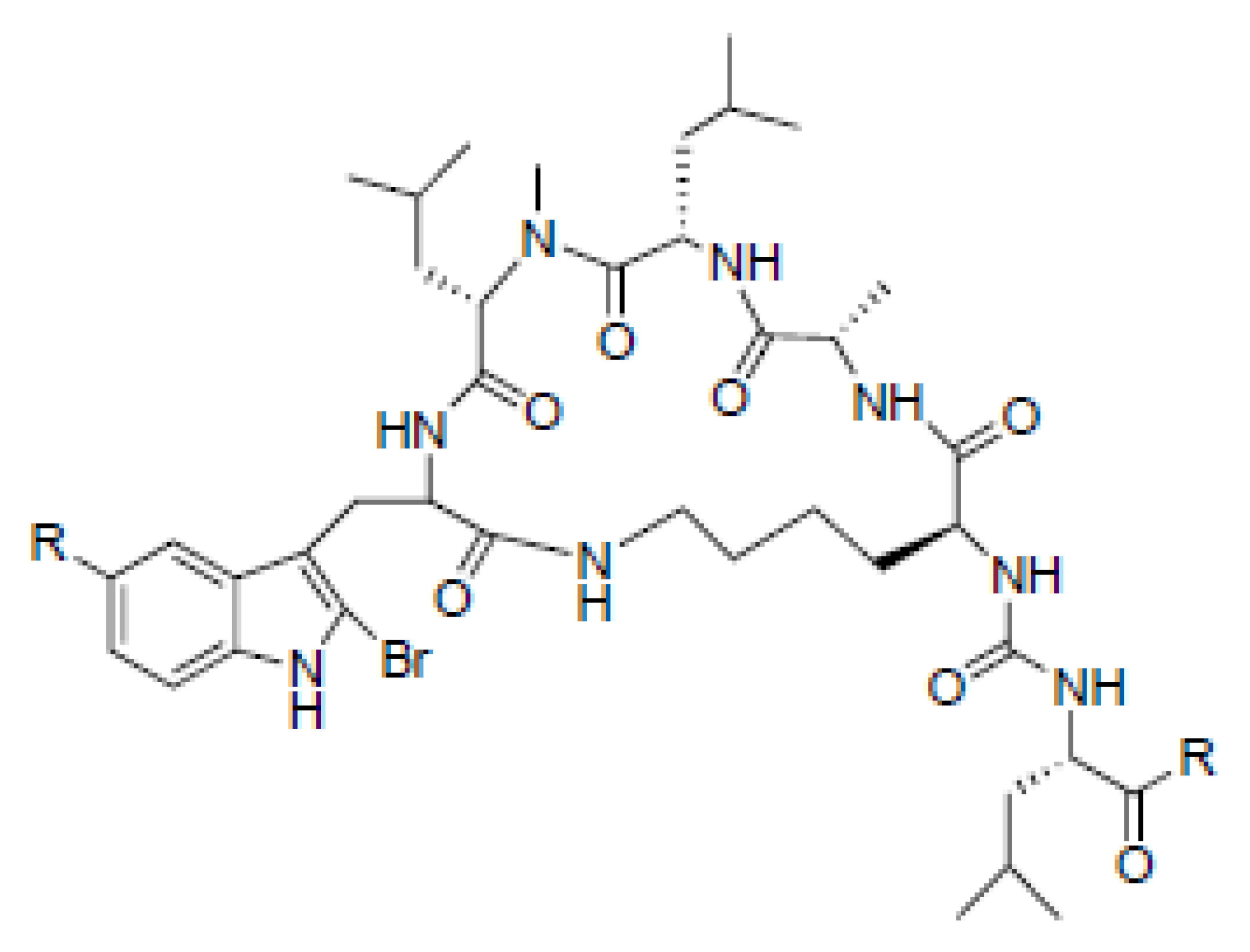 R: -OH; -OMe	2-Bromo-5-hydroxytryptophan	*Theonella* spp.	[70]
**Jaspamide**	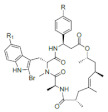 R: OH; OAc; R_1_: H Jaspamide R2: Br Jaspamide R	N-Methyl-2-bromotryptophan N-Methyl-2,5-bromotryptophan	*J. spendens*	[71]
**Jasplakinolide**	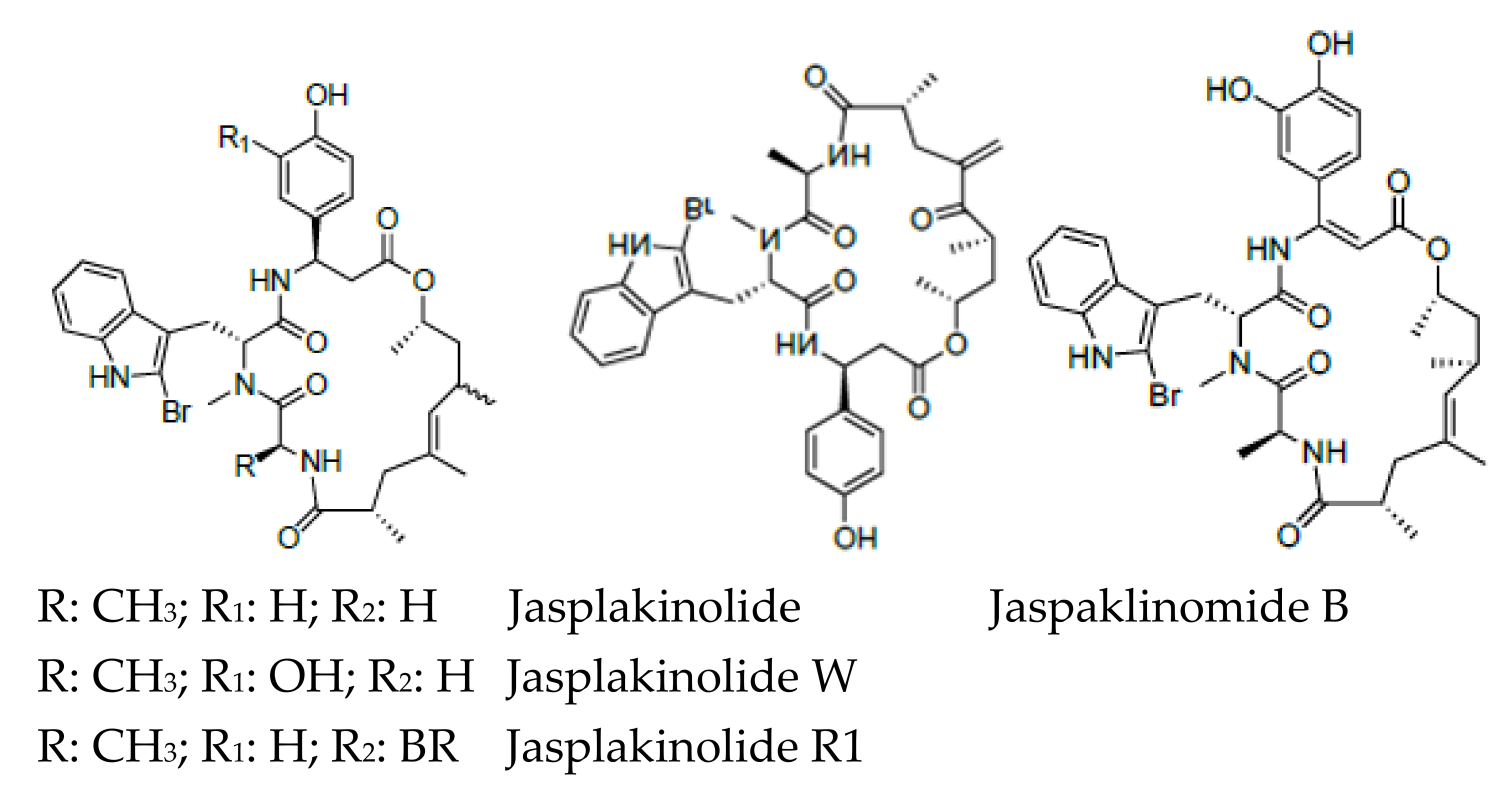	2-Bromotryptophan	*J. spendens*	[93,94]
**Cyclocinamide A**	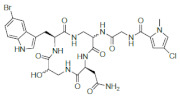	5-Bromotryptophan	*Psammocinia* spp.	[95]
**Barettin**	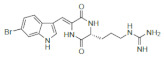 Barettin 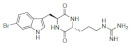 8,9-Dihydrobarettin 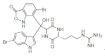 Bromobenzisoxazolo	5-Bromotryptophan 6-Bromotryptophan	*G. barretti*	[96] [97]
**Halocyamine**	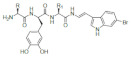 R: His; R1: Gly; Halocyamine A R: Thr; R1: His; Halocyamine B	6-Bromo 8,9 didehydro- Tryptamine	*H. roretzi*	[98]
**Eusynstyelamide 1**	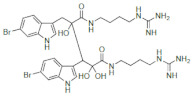	6-Bromotryptophan	*E. misakiensis*	[96] [97]
**Eusynstyelamide A**	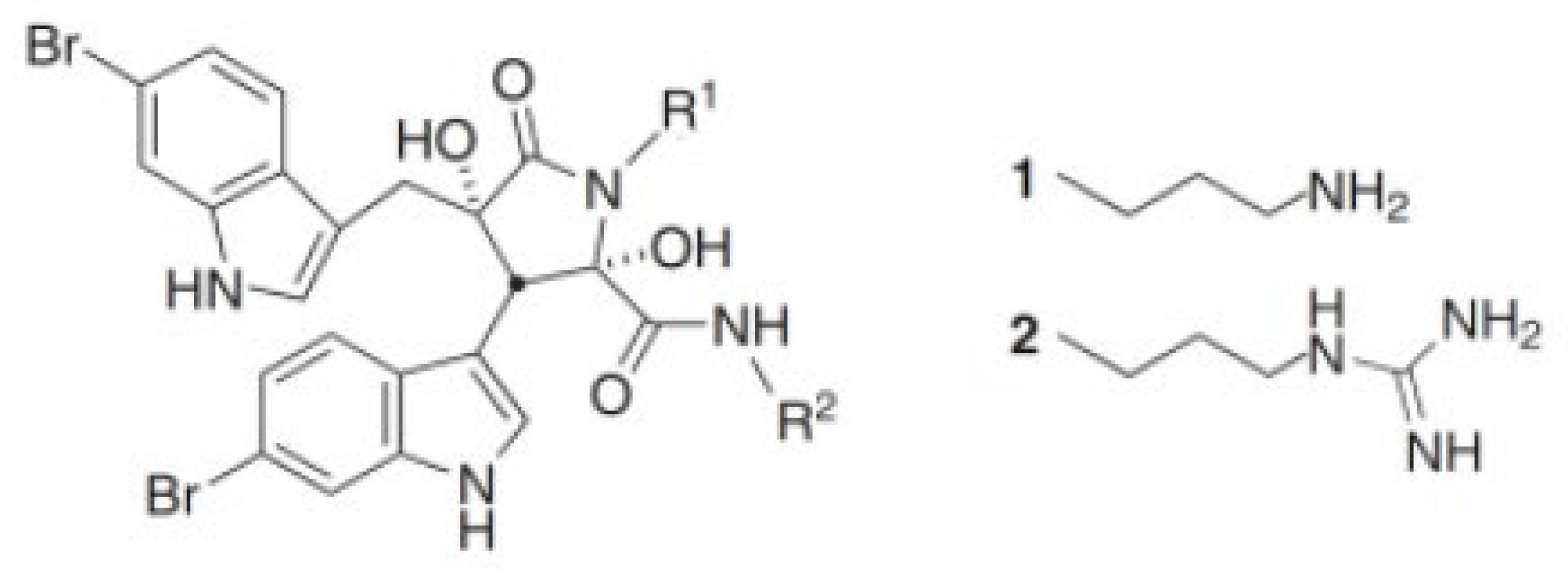 R_1_: **2**; R_2_: **2** Eusynstyelamide A-C (enantiomers) R_1_: **1**; R_2_: **1** Eusynstyelamide D R_1_: **2**; R_2_: **1** Eusynstyelamide E R_1_: **1**; R_2_: **2** Eusynstyelamide F	6-Bromotryptophan	*E. latericius*	[98]
**Apliamide**	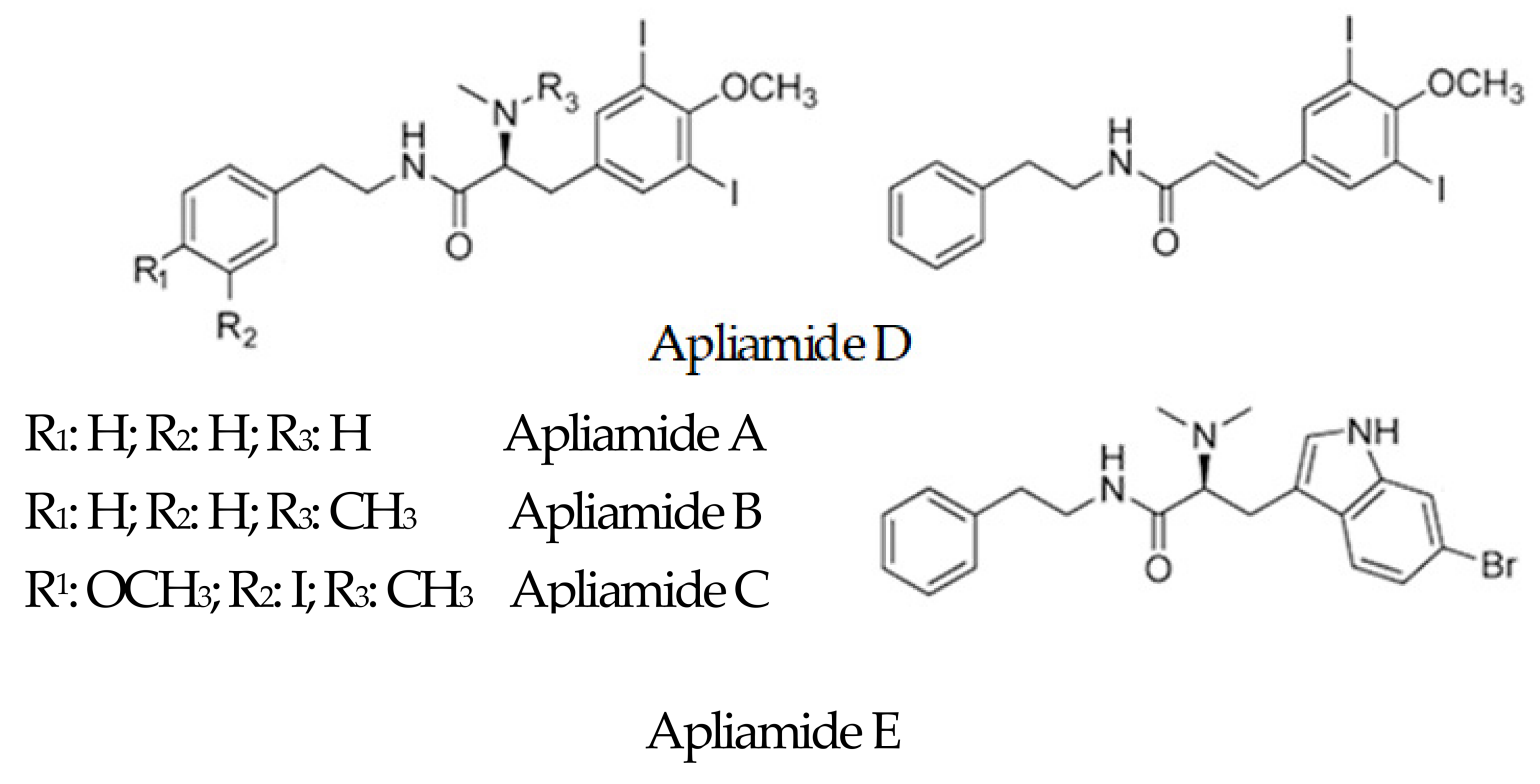	6-Bromotryptophan 3, 5-Iodio-4-methoxytyrosine	*Aplidium* spp.	[63]
**Valviamides**	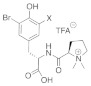 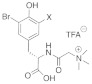 X = Br Valviamide A (**11**) X = Br Valviamide C (**13**) X = I Valviamide B (**12**) X = I Valviamide D (**14**)	3,5-Iodiotyrosine 3,5-Bromotyrosine	*A. hickmani* *P. darwini*	[65]

**Table 3:** Gene-encoded, ribosomally synthesized peptides cited in the review (adapted from Ref. [92]).

Name	Sequence ^a^	Halo ^b^	Source	Ref.
**HFIAP1** **HFIAP2** **HFIAP3**	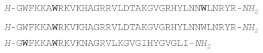	**W:** 6-BrTrp	Hagfish: *M. glutinosa*	[165]
**STYELIN D**	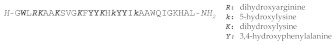	**W:** 6-BrTrp	Ascidian: *S. clava*	[166]
**HEDISTIN**	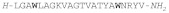	**W:** 6-BrTrp	Annelid: *N. diversicolor*	[167]
**STRONGYLOCIN 2**	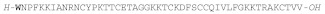	**W:** 6-BrTrp	Sea urchin: *S. droebachiensis*	[168]
**EeSTRONGYLOCIN 2**	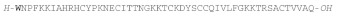	**W:** 6-BrTrp	Sea urchin *E. esclulentus*	[169]
**EeCENTROCIN 1**	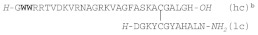	**W:** 6-BrTrp	*E. esclulentus*	[169]
**EeCENTROCIN 2**	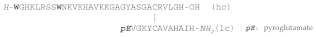	**W:** 6-BrTrp	*E. esclulentus*	[169]
**SdCENTROCIN 1**	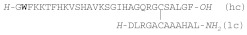	**W:** 6-BrTrp	*S. droebachiensis*	[170]
**SdCENTROCIN 2**	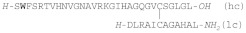	**W:** 6-BrTrp	*S. droebachiensis*	[170]

^a^ modified amino acids are in bold. ^**b**^ hc: heavy chain; lc: light chain.

## Data Availability

Not applicable.
